# Ubiquitin carboxyl-terminal esterase L1 (UCHL1) is associated with stem-like cancer cell functions in pediatric high-grade glioma

**DOI:** 10.1371/journal.pone.0176879

**Published:** 2017-05-04

**Authors:** Patricia C. Sanchez-Diaz, Judy C. Chang, Emily S. Moses, Tu Dao, Yidong Chen, Jaclyn Y. Hung

**Affiliations:** 1 Greehey Children’s Cancer Research Institute, University of Texas Health Science Center San Antonio, San Antonio, Texas, United States of America; 2 Rosenberg School of Optometry, University of the Incarnate Word, San Antonio, Texas, United States of America; 3 Department of Pediatrics, University of Virginia, Charlottesville, Virginia, United States of America; 4 Epidemiology and Biostatistics, University of Texas Health Science Center San Antonio, San Antonio, Texas, United States of America; 5 Division of Hematology and Oncology, Department of Pediatrics, University of Texas Health Science Center San Antonio, San Antonio, Texas, United States of America; Universidad de Navarra, SPAIN

## Abstract

Pediatric high-grade gliomas represent 8–12% of all primary tumors of the nervous system in children. Five-year survival for these pediatric aggressive tumors is poor (15–35%) indicating the need to develop better treatments for pediatric high-grade gliomas. In this work we used SF188 and SJ-GBM2 cell lines to study the function of the ubiquitin carboxyl-terminal esterase L1 (UCHL1), a deubiquitinase de-regulated in several cancers, in pediatric high-grade gliomas. UCHL1 depletion in SF188 and SJ-GBM2 glioma cells was associated with decreased cell proliferation and invasion, along with a reduced ability to grow in soft agar and to form spheres (i.e. self-renewal measure). A 70% reduction in Wnt signaling was also observed in the SF188 and SJ-GBM2 UCHL1 knockdowns (KDs) using a TCF-dependent TOPflash reporter assay. Transcriptome comparisons of UCHL1 KDs versus vector control identified a list of 306 differentially expressed genes (at least 2-fold change; *p* <0.05) which included genes known to be involved in cancer like *ACTA2*, *POSTN*, *LIF*, *FBXL7*, *FBXW11*, *GDF15*, *HEY2*, but also potential novel genes such us *IGLL5*, *ABCA4*, *AQP3*, *AQP4*, *CALB1*, and *ALK*. Bioinformatics gene ontology (GO) analysis of these 306 genes revealed significant enrichment in “signal peptides”, “extracellular matrix”and “secreted proteins” GO Terms. “Angiogenesis and blood vessel development”, “neuron differentiation/development”, cell adhesion”, and “cell migration” also showed significant enrichment in our GO analysis. Top canonical pathways identified by Ingenuity Pathway Analysis (IPA) included “Clathrin-mediated Endocytosis Signaling” (*p* = 5.14x10^-4^), “Virus Entry via Endocytic Pathways” (*p* = 6.15x 10^−4^), and “High Mobility Group-Box 1 (HMGB1) Signaling” (*p* = 6.15x10^-4^). While FGF2, IL1B, TNF and PDGFB were predicted as top upstream regulators (*p* < 2x10-^16^) of the UCHL1 KD-associated transcriptome. Aberrant expression of UCHL1 in pediatric high-grade gliomas may promote cell invasion, transformation, and self-renewal properties, at least in part, by modulating Wnt/Beta catenin activity. UCHL1 might act as an oncogene in glioma within the gene network that imparts stem-like characteristics to these cancer cells.

## Introduction

Childhood high-grade astrocytoma, although rare, is extremely difficult to treat. For a vast majority of the patients, the disease quickly relapses following an initial response to aggressive multimodality treatments and about 80% of the affected children will die from their disease [1]. Malignant gliomas show substantial genetic and histological heterogeneity, which supports the notion of pathway pliability [2]. The clinical presentations, which includes invasion of surrounding tissue, and the response of these patients to therapy supports the concept that a pool of stem-like cancer cells responsible for long-term remission failure that exits within these tumors [3–5]. In this scenario, identifying pathways that impart stem-like characteristics to glioma cells can offer radical advances to the treatment and diagnosis of this devastating childhood cancer.

Ubiquitin carboxyl-terminal esterase L1 (UCHL1) is a deubiquitinating enzyme (DUB) of the ubiquitin proteasome system (UPS), the major cellular machinery that regulates protein homeostasis. The UPS is responsible for intracellular protein degradation and regulation of many key biological processes, such as breakdown of transcription factors, cell cycle control, and cell differentiation [6, 7]. Defect in the ubiquitin-proteasome pathway is observed in several human diseases including neurodegenerative diseases [8, 9] and in certain types of malignant tumors [10, 11]. In this regard, UCHL1 is differentially expressed in various cancers, and has been proposed to have oncogenic or tumor suppressive properties depending on the cellular context [12]. UCHL1 activity in tumorigenesis has been linked to cell cycle regulation, possibly, by targeting p53, β-catenin, and Akt pathways [13–16]. UCHL1 has been shown to promote metastasis via the activation of HIF-1 and its overexpression also correlates with poor prognosis in patients with breast and lung cancers [17].

Several lines of evidence suggest that UCHL1 is essential for the onset of neurogenesis and that is a determinant of asymmetric distribution during germ-line stem cell self-renewal and differentiation [18, 19]. Data from the Protein Atlas Database (http://www.proteinatlas.org/), shows a distinct cytoplasmic and nuclear UCHL1 immunoreactivity in glioma samples. Although the clinical relevance of UCHL1 expression in glioma seems possible, whether UCHL1 overexpression contributes to the malignant transformation/phenotype in astrocytoma has not been ascertain, and the molecular mechanism underlying its action in this context is also unclear. The ubiquitin proteasome system has emerged as a promising target for cancer therapy with two drugs targeting the proteasome (bortezomib and carfilzomib) currently approved by the FDA for the treatment of multiple myeloma. Improved oral bioavailability and specificity of action together with targeting “undruggable” oncoproteins (e.g. MYCN, Beta-catenin) are areas of interests in cancer therapy research (reviewed in [20]). Therefore, DUB enzymes found to be deregulated in cancer, such UCHL1, may give rise to alternative cancer therapies as upstream regulators of “undruggable” oncoproteins. Also, increased selectivity of DUB enzymes over the currently FDA approved proteasome inhibitors is a driving force for exploring DUBs as potential anticancer targets.

Using a lentiviral knockdown system, we investigated the effect of UCHL1 inhibition on astrocytoma cell invasion, cell proliferation, and on the stem-like cancer cell population. To gain some insight in the UCHL1-associated pathways, we then conducted transcriptomic and bioinformatics analyses with the control and knockdown cell lines. Our loss of function studies using these pediatric high-grade gliomas cell lines showed that UCHL1 promoted cell growth, invasiveness, and self-renewal characteristics *in vitro*. Using a luciferase reporter system, we found significant downregulation of Wnt/Beta catenin signal transduction in the UCHL1 KDs, which suggested this pathway as a possible candidate mediating, at least in part, the observed effects. Subsequent transcriptomic and bioinformatics analyses identified angiogenesis, neuron development and differentiation, or cell motility among the potential functions of the UCHL1-associated gene network. Endocytosis, chemokine signaling, and extracellular matrix-receptor interaction were also identified in our model system as possible pathways associated to the UCHL1-KD transcriptome.

## Results

### UCHL1 is highly expressed in high-grade glioma cell lines and tumor samples

To gain some insight in the potential role of UCHL1 in high-grade glioma, we mined its levels of expression in human glioma tissue microarrays reported in the publicly available Protein Atlas Database; http://www.proteinatlas.org/. The expression levels of UCHL1 in these tissue microarray glioma samples from the database is summarized in Table 1.

**Table 1:** UCHL1 is expressed in high-grade glioma samples reported in the Protein Atlas Database; http://www.proteinatlas.org/.

Glioma type	UCHL1 antibody staining			
	High^a^	Medium^a^	Low^a^	Not detected^a^
High-grade (pediatric)	1	1	--	--
High-grade (adult)	5	3	5	3
Low-grade (adult)	2	1	1	--

^a^ Number of glioma samples in each category (http://www.proteinatlas.org/; retrieved on March 14, 2017).

**Fig 1:**
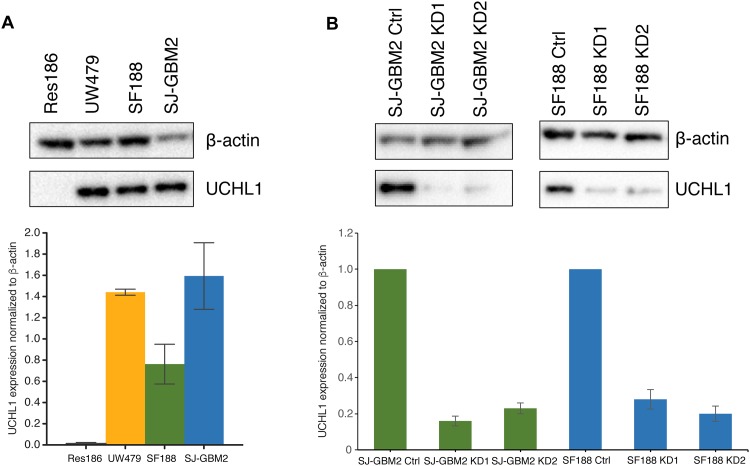
UCHL1 expression levels in pediatric glioma cell lines. A) UCHL1 protein expression is elevated in pediatric high-grade glioma UW479, SF188 and SJ-GBM2 cells compared to low grade Res186 cells. Quantified data are presented as mean densitometry values ±SEM (n = 3); *P ≤* 0.05. Representative blot demonstrates non detectable expression of UCHL1 in Res186 protein extracts at the exposure times that resulted in band immunoreactivity saturation in the high-grade gliomas. B) Using a lentiviral infection system UCHL1 knockdowns (KDs) were generated in SF188 and SJ-GBM2 cells. At least a 70% reduction in UCHL1 was obtained at the level of protein and gene expression as determined by western blot and digital droplet PCR and confirmed by RNAseq analyses. Representative western blots are shown for the SJ-GBM2 and SF188 knockdowns. Quantified data are presented as mean densitometry values ±SEM (n = 3); *P ≤* 0.05 normalized to β-actin and relative to control SJ-GBM2 and SF188 cells infected with the shTurboGFP vector.

This tissue microarray consists of 20 adult and 2 childhood glioma samples. A distinct cytoplasmic and nuclear UCHL1 immunoreactivity was reported in 19 of the 22 samples in the microarray which included the two pediatric cases and 13 of 16 high-grade adult astrocytoma cases (http://www.proteinatlas.org/). Then, using well-characterized pediatric astrocytoma cell lines as a model system, we observed elevated UCHL1 mRNA expression with correlating protein expression in UW479, a WHO grade III anaplastic astrocytoma, SF188, and SJ-GBM2, WHO grade IV glioblastoma mutiforme cell lines but no expression in Res186, a WHO grade I pilocytic astrocytoma cell line. The mean densitometry values with SEM of three western blots along with the digitalized image of one of the blots is shown in Fig 1A. Thus, the tissue microarray and cell lines data suggested a possible role for UCHL1 in the higher grade glioma cancer cells.

### UCHL1 promotes *in vitro* clonogenicity, cell proliferation, and invasion

To explore the relevance of UCHL1 in glioma growth and invasion, we carried out several *in vitro* analyses using a loss of function strategy. We knocked down UCHL1 expression in SF188 and SJ-GBM2 cells utilizing two different shRNA constructs (herein called UCHL1 KD1 and KD2). At least a 70% reduction in *UCHL1* transcript levels as measured by ddPCR and confirmed by RNAseq was achieved in all the four KDs compared to their shRNA controls. The mean and SEM densitometry values for UCHL1 protein levels, and their representative western blots in the SJ-GBM2 and SF188 knockdowns are shown in Fig 1B. As shown in this figure, UCHL1 expression was knocked down to 0.16 ± 0.03 and 0.23 ± 0.03 in SJ-GBM2 KD1, and SJ-GBM2 KD2 respectively, and to 0.28 ± 0.05 and 0.20 ± 0.04 in the SF188 KD1, and SF188 KD2 respectively.

**Fig 2:**
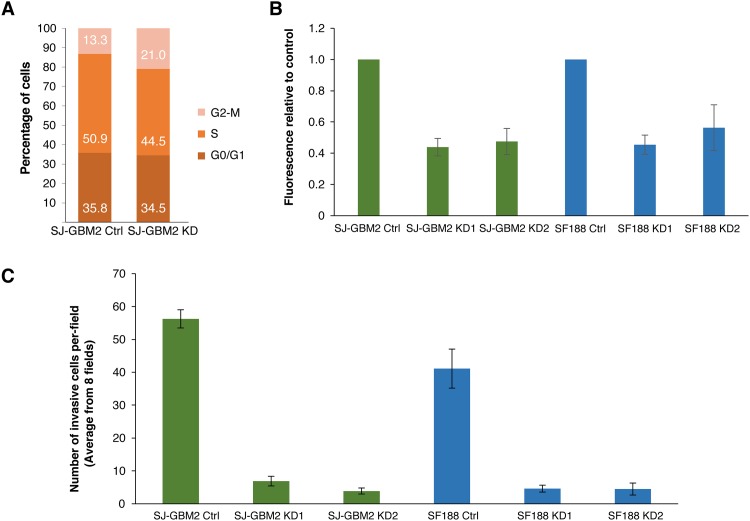
UCHL1 promotes cell proliferation and invasion. A) Cell cycle distribution. UCHL1 depletion is associated with reduction on the percentage of SF188 cells in S phase (from 50.9% in the control to 44.5% in the KD) and a parallel increase in the fraction of cells on G2/M (from 13.3% to 21%). B) Soft agar growth. CytoSelect 96-Well Cell Transformation Assay, Soft Agar Colony Formation (Cell Biolabs) was used to assess anchorage independent growth. Cells were incubated for 10 days. On average, a 40–50% decrease in soft agar growth was observed in the UCHL1 knockdowns compared to control SJ-GBM2 and SF188 cells (*P ≤* 0.05). Data are shown as mean ± SEM values (n = 3; performed in duplicates). C) Cell invasion was measured in control and UCHL1 knockdowns using CytoSelect 24-Well Assay (Cell Biolabs). The average number of invasive cells from eight fields after 24 hours of incubation is shown as mean values ± SEM (n = 3; performed in duplicates). At least a 7-fold decrease in cell invasiveness was observed in the knockdowns (*P*< 0.001). Per field, on average 56 (56.26 ± 2.77) control SJ-GBM2 cells were able to migrate through the Matrigel substrate, whereas only ~7 (6.9 ± 1.46) and ~4 (3.9 ± 0.912) were able to invade in the knockdowns. Similar results were observed in the case of SF188 glioma cell with the number of invasive cells dropping from ~40 (41.12 ± 5.94) in the controls to ~5 in the knockdowns (4.6 ± 1.051 and 4.5 ± 1.82 for KD1 and KD2 respectively).

To assess the role UCHL1 *in vitro* tumorigenicity, a colony formation assay (ie, soft-agar growth assay) was used. As shown in Fig 2B, close to 50% reduction in clonogenicity was observed in the SF188 and SJ-GBM2 UCHL1 KDs, suggesting a functional role of UCHL1 in glioma malignant transformation. The ability of forming colonies in soft agar was 0.45 ± 0.06 and 0.56 ± 0.15 for SF188 KD1 and KD2 respectively, and 0.44 ± 0.07 and 0.47 ± 0.08 for SJ-GBM2 KD1 and KD2, respectively.

Table 2 shows that depletion of UCHL1 increased the population doubling times to 50–70 hours, suggested that UCHL1 might play a role in promoting cell proliferation. To elucidate the possible role of UCHL in cell proliferation, cell cycle analysis was performed. As shown in Fig 2A, depletion of UCHL1 increased the percentage of cells in the G2/M phases of the cell cycle from 13% in the control cells to 21% in the UCHL1 knockdowns.

**Table 2:** UCHL1 knockdowns showed increased cell doubling times.

Cell Line	Doubling Time^a^		
	Control	UCHL1 KD1^b^	UCHL1 KD2^b^
**SJ-GBM2**	32	73	52
**SF188**	29	75	51

^a^In hours.

^b^Two different shRNA constructs.

A key feature of malignant gliomas is their ability to migrate and to invade normal surrounding tissue. Invasive cells produce proteolytic enzymes that enable them to detach from their basement membrane, to digest the extracellular matrix, and to migrate. To investigate possible differences invasiveness between the knockdowns and controls, an *in vitro* Matrigel assay was done. Fig 2C shows that UCHL1 depleted cells were significantly less invasive than the control cells. Approximately, a 90% decrease in the number of cells able to migrate through the basement membrane inserts was found in the knockdown samples. We scored the number of cells invading 8 fields (magnification, 40x) of the Matrigel inserts of the controls and knowndowns. The average number of invasive cells in the 8 fields of the Matrigel were: 7 in the case of SJ-GBM2 KD1, 4 in SJ-GBM2 KD2 cells, 5 in SF188KD1, and 5 in SF188 KD2 compared to 56 in the SJ-GBM2, and 40 in the SF188 control cells, respectively. Thus our cell-based assays suggested a potential role of UCHL1 in glioma cell transformation and cell invasiveness.

### UCHL1 promotes self-renewal in glioma stem-like cells

**Fig 3:**
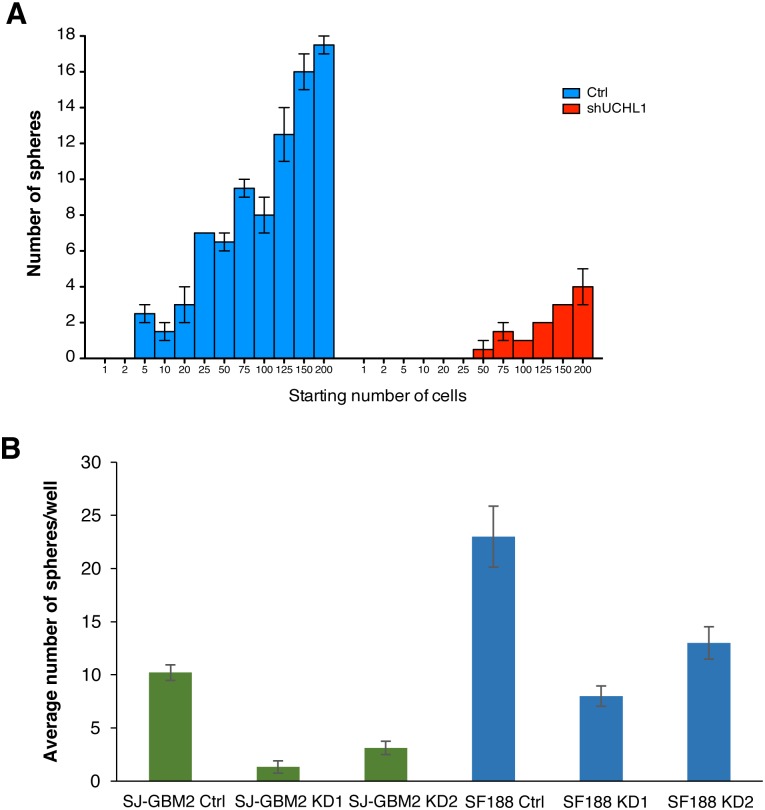
Sphere formation in impaired in the UCHL1 knockdowns. A) Sphere dilution assay. Second generation of control and UCHL1 knockdown spheres were dispersed and re-plated in ultra-low attachment 96-well plates using serial dilution (1 to 200 cells/well range) to assess sphere formation efficiency after 10 days in culture. On average, 10% of SJ-GBM2 control cells were able to form spheres compared to 2% in the case of the UCHL1 knockdown. B) SJ-GBM2 and SF188 control and KD second generation of spheres were dispersed and plated at a density of 2x10^3^ cells/mL. The number of third generation spheres was scored after seven days of incubation. Scored data is shown as mean number of spheres larger than 50 μM in diameter ± SEM (n = 3; performed in triplicates); *P ≤* 0.05.

Our data suggested that UCHL1 might affect glioma growth and invasiveness, these are known properties associated with stem-like cell populations. Spheroid cultures have been demonstrated to enrich in glioblastoma stem-like cancer cells and are considered a surrogate marker of self-renewal capability [21]. Using this well-established protocol for glioma CSC culture, first we determine the optimum cell density at plating so that cells were dispersed to ensure that each third generation sphere is derived from a single cell, which is a requirement to assess self-renewal *in vitro*. As shown in Fig 3A, we determined that plating cell densities from 50 to 200 cells per well of a 96-well plate provided a reproducible results, with a sphere formation rate of ~10% in the control and ~2% in the UCHL1 KD. As shown in Fig 3B, there was an approximately 60% reduction in the number of third generation spheres in the SF188 KDs (i.e. 8 ± 1 and 13 ± 1 in the KD1 and KD2 compared to 23 ± 3 in the control shRNA construct). A 80% reduction was observed in the SJ-GBM2 KDs (i.e. 1.3 ± 0.6 and 3.1 ±0.6 in the KD1 and KD2 versus 10.2 ± 0.7 in the control shRNA construct). These results suggested a potential role of UCHL1 in pediatric high-grade glioma stem-like cells.

### Predicted cellular pathways associated to the UCHL1-KD transcriptome

De-regulation of p53, β-catenin, and Akt pathways have been linked to the activity of UCHL1 in tumorigenesis [13–16]. In this regard, UCHL1 has been proposed to form a positive feedback loop with β-catenin, whereby UCHL1 promotes translocation of β-catenin into the nucleus where it binds to the TCF/LEF complex and activates transcription of its downstream targets, which may also include *UCHL1* [22]. Furthermore, abnormal of Wnt/Beta-catenin signaling has been reported in glioma stem-like cancer cells [23]. Thus, we sought to determine whether UCHL1 was involved in Wnt/Beta-catenin activity in our model system.

**Fig 4:**
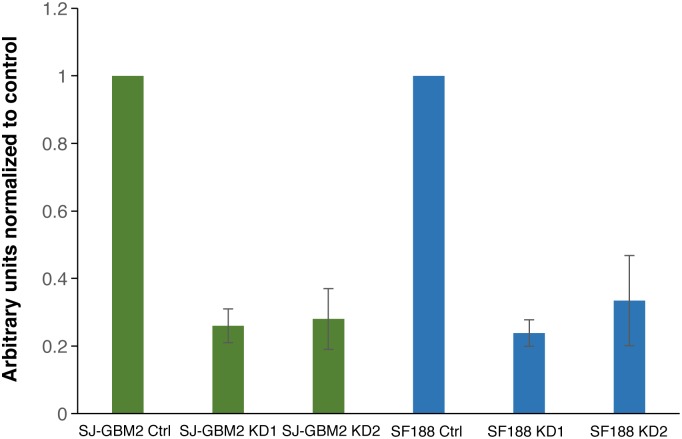
UCHL1 depletion decreases Wnt signaling activity. Using a TCF-dependent TOPflash reporter assay, on average, a 70% reduction in the activity of the Wnt pathway was found in the UCHL1 KDs compared to controls. Relative to control cells the Wnt signaling activity in the SJ-GBM2 knockdowns was decrease to 0.26 ± 0.05 (KD1) and 0.28 ± 0.09 (KD2) and, to 0.24 ± 0.04 (KD1) and 0.34 ± 0.13 (KD2) in the case of SF188 knockdowns. Data is presented as mean ± SEM (n = 3); *P ≤* 0.05

We used a TOPflash luciferase reporter assay to assess Beta-catenin-mediated activation of downstream target genes in our model system. We found, on average, a 70% decrease in Wnt/Beta-catenin signaling when UCHL1 was depleted. As shown in Fig 4, the mean and SEM arbitrary units of luminescence were 0.26 ± 0.05 (SJ-GBM2 KD1), 0.28 ± 0.09 (SJ-GBM2 KD2), 0.24 ± 0.04 (SF188 KD1), and 0.34 ± 0.13 (SF188 KD2), normalized to their respective control cells. These data suggested the beta-catenin pathway as a mediator, at least in part, of the effects of UCHL1 in SJ-GBM2 and SF188 glioma cells.

To obtain additional insights in the UCHL1-associated gene networks and potential mechanisms mediating the UCHL1 effects, we performed transcriptome comparisons of the cells transduced with the UCHL1 shRNA constructs and those transduced with the shRNA control plasmid. Three hundred and six differentially expressed genes were found by RNAseq in the SJ-GBM2 UCHL1 knockdowns and control cells (S1 File), the top 32 differentially expressed genes are listed in Table 3.

**Table 3:** UCHL1 knockdown-associated transcriptome.

Down-regulated genes	Log_2_ fold-change^a^	Up-regulated genes	Log_2_ fold-change^a^
*IGLL5*	-5.329	*SERPINB4*	5.957
*AQP4*	-5.213	*TAC1*	5.432
*ABCA4*	-4.766	*FGF21*	4.621
*GRAP2*	-4.748	*ANKRD1*	4.197
*FAM83F*	-4.457	*ENPP2*	4.137
*SP7*	-4.239	*SBSN*	4.054
*CALB1*	-4.187	*GDF15*	3.737
*PTPN22*	-3.940	*C16orf73*	3.703
*TCA3*	-3.854	*NGFR*	3.643
*PDE7B*	-3.764	*MT1G*	3.566
*EDN3*	-3.675	*HMOX1*	3.442
*AMBN*	-3.570	*SOCS2*	3.332
*DLK1*	-3.493	*KCNS3*	3.402
*KLHL4*	-3.461	*MTE1*	3.104
*CTLA4*	-3.428	*MT1X*	3.082
*OGN*	-3.392	*NUPR1*	3.030

^a^Knockdown versus control (*p* < 0.05).

*In silico* gene ontology (GO) analysis of these 306 differentially genes identified 11 annotation clusters with enrichment scores above 2.5 (DAVID, [24]). Table 4 summarizes the top GO annotated clusters and biological processes identified in the bioinformatics analyses.

**Table 4:** Top annotated clusters and biological processes identified by gene ontology in the UCHL1 knockdowns.

Top annotation clusters and biological processes^a^			
Name	Cluster Score	*P*-value	Genes (N = 306)^b^
*Signal Peptides*, *Extracellular Region*	8.33	4.07x10^-11^	97
*Glycosylation*, *Disulfide Bond*	6.87	1.13x10^-6^	98
*Extracellular Matrix*	3.65	8.31x10^-5^	18
*Plasma Membrane proteins*	3.61	4.33x10^-5^	40
*Angiogenesis*	2.89	8.77x10^-4^	11
*Neuron differentiation*	2.79	8.95x10^-8^	26
*Cell adhesion*	2.65	8.62x10^-4^	14
*Cell migration*	2.56	5.41x10^-6^	14

^**a**^ DAVID Bioinformatics Resources 6.8: https://david.ncifcrf.gov/

^b^ Number of differentially expressed genes found in the annotated GO Term.

The top 4 clusters (with enrichment scores 8.33, 6.87, 3.65, and 3.61 respectively) were related to “signal peptides”, “extracellular matrix“, “secreted proteins”, and “plasma membrane (proteins)” GO Terms (S2 File). Specifically, top enriched GO Terms identified with this analysis were “signal peptide” (with 97 genes involved; 32.33% enrichment; *p* = 4.07x10^-11^), extracellular region” (63 genes; 21% enrichment, *p* = 9.78x10^-7^), “glycoprotein” (103 genes; 34% enrichment; *p* = 1.13x10^-6^), and “integral to plasma membrane” (40 genes; 13.33% enrichment; *p* = 4.33x10^-5^). Additional biological process showing significant cluster enrichment in the GO analysis included “angiogenesis and blood vessel development”, (enrichment score 2.89) “neuron differentiation/development” (2.79), “cell adhesion” (2.65), and “cell migration” (2.56). Thus these GO analyses seemed to suggest a potential role for UCHL1 in extracellular matrix composition or extracellular matrix-cell communication. Intriguingly, our Ingenuity Pathway Analysis (IPA) also identified, inflammation, endocytosis, metastasis related functions and “High Mobility Group Box-1 (HMGB1) Signaling” within the top canonical pathways possibly affected by UCHL1 in SJ-GBM2 cells (i.e. “Role of Macrophages, Fibroblasts and Endothelial Cells in Rheumatoid Arthritis”; *p* = 3.68x10^-5^, “Clathrin-mediated Endocytosis Signaling”; *p* = 5.14x10^-4^, “Virus Entry via Endocytic Pathways”; *p* = 6.15x10^-4^, “HMGB1 Signaling”; *p* = 6.15x10^-4^, “Colorectal Cancer Metastasis Signaling”; *p* = 8.18x10^-4^), also in line with a possible role of UCHL1 in cell-extracellular matrix interaction. These UCHL1-associated pathways and upstream regulators are summarized in Table 5.

**Table 5:** UCHL1-associated pathways.

Top Canonical Pathways and Upstream Regulators^a^	
Name of Pathway or Regulator	*P-*value
*Role of Macrophages*, *Fibroblasts and Endothelial Cells*	3.68x10^-5^
*Clathrin-mediated Endocytosis Signaling*	5.14x10^-4^
*Virus Entry via Endocytic Pathways*	6.15x10^-4^
*HMGB1 Signaling*	6.15x10^-4^
*Colorectal Cancer Metastasis Signaling*	8.18x10^-4^
*FGF2* (upstream regulator)	3.57x10^-18^
*IL1B* (upstream regulator)	7.67x10^-18^
*TNF* (upstream regulator)	1.04x10^-16^
*PDGF BB* (upstream regulator)	1.95x10^-16^
*Dexamethasone* (upstream regulator)	3.82x10^-16^

^a^IPA version: 389077M. Content version: 27821452 (released on 2016-06-14).

The top three potential upstream regulators of the UCHL1-KD transcriptome predicted by IPA software (S3 File) were coagulation factor II (*F2*, *p* = 5.96x10^-9^; with 18 targets in our differentially expressed genes), nuclear protein 1 (*NUPR1*, *p* = 1.17x10^-4^; with 18 target genes), and P53 (*TP53; p* = 2.14x10^-15^; with 58 target genes). Of note, expression of *NUPR1* (a transcription factor involved in progression of liver and other cancers) was about 8-fold increased in our UCHL1 knockdown (*p* = 1.20x10^-8^).

Within the list of top differentially expressed genes, in the UCHL1 KDs we found downregulation of the Wnt targets *POSTN*, *SP7*, and *DLL1*, of the transporter *ABCA4*, of the homeobox gene *DLX4*, and of genes recently linked to cancer progression *ACTA2*, *AQ4*, *GRAP2*, and *ALK*.

## Discussion

Tumorigenesis is a complex process that involves abnormal changes in the pathways mediating cell proliferation, survival, and motility. Cancer cells usually achieve these functions by hijacking developmental pathways including Wnt/Beta-catenin, Sonic Hedgehog, Akt or Notch to name some. High-grade gliomas are very heterogeneous tumors that may resemble a developmental program going awry with glioma cancer stem cells positioned at the top of the hierarchy. These cancer stem cells are defined operationally in analogy to normal adult stem cell, as cells with the dual capacity to regenerate themselves through self-renewal mechanism and to perpetuate the production of phenotypically heterogeneous progeny with limited proliferative ability [25].

High-grade gliomas are the most aggressive type of primary brain tumors and their great tendency to invade surrounding tissue represents a real therapeutic challenge [1, 26]. Cancer cells with stem-like properties have been identified in high-grade gliomas and have been proposed as the basis for the plasticity and survival of these malignant tumors. These CSCs are able to regenerate themselves, to differentiate into the cells forming the bulk of the tumor, and to communicate with their microenvironment to increase angiogenesis or to recruit inflammatory cells that sustain tumor growth and survival [27]. Thus, novel strategies in high-grade gliomas, and in many other types of cancers, aim to target these CSC populations is warranted.

UCHL1 is a deubiquitinase highly expressed in the nervous system and its role in different types of cancer is starting to be elucidated. Recent studies have demonstrated that UCHL1 can affect invasion, epithelial-mesenchymal transition, chemosensitivity, and cell cycle among other functions [14, 28–30]. The available protein expression data from tumor samples (Protein Atlas database; http://www.proteinatlas.org/) supported a potential role of UCHL1 in high-grade glioma. Using cell lines as model system we confirmed that UCHL1 was highly expressed in high-grade pediatric glioma SF188 and SJ-GBM2 cells compared to low-grade Res186 cells. As shown in Fig 1A, SJ-GBM2 cells exhibited higher levels of UCHL1 protein compared to SF188 and UW479 cells. This data provided us with the rationale to explore whether UCHL1 was involved in high-grade glioma malignancy. Future work will explore the biological relevance of the differences in UCHL1 expression among the various WHO grade astrocytomas.

In this study, we knocked down the expression of UCHL1 in SF188 and SJ-GBM2 cell lines using a lentivirus transduction system of two different UCHL1 shRNA vectors, providing us a model system to elucidate the potential function of UCHL1 in glioma malignancy. We assessed the effects of UCHL1 depletion upon sphere formation, cell invasion, and cell proliferation. A decrease on cell proliferation and in cellular invasiveness, together with an impaired sphere formation (*in vitro* surrogate assay for self-renewal), was observed in the knockdowns, which suggested a potential role for UCHL1 in high-grade glioma CSC function.

Although activating mutations in beta-catenin do not seem frequent in gliomas, intracellular accumulation of beta-catenin and Wnt pathway activation occur in glioblastoma and they contribute to CSC maintenance and cell invasion [31]. For example, PLAGL2 (Pleomorphic adenomas gene-like 2) and Dishevelled-2 (DVL2) have been shown to activate Wnt pathway in glioblastomas and to sustain self-renewal in glioma CSCs independently of beta-catenin mutations [23, 32]. UCHL1 has also been proposed to activate Wnt signaling through deubiquitination and stabilization of beta-catenin [33]. In this study, using TCF-dependent TOPflash reporter system we found approximately, a 70% decrease in the activity of Wnt pathway in our KDs model system, thus suggesting that Wnt or of its downstream effectors could be influencing sphere formation, cell proliferation, and/or invasion. Of note, in the UCHL1 KDs we found a marked decrease in the gene expression of the Wnt targets *SP7* (a transcription factor involved in osteoblast differentiation; ~19-fold decrease), *DLL1* (delta like canonical Notch ligand 1; ~11-fold decrease), and *POSTN* (~6-fold decrease), thus consistent with a reduced activity of the pathway in these cells. *POSTN* gene encodes for periostin; an extracellular protein secreted by glioma cancer stem cells that recruits macrophages to the CSC niche, promotes angiogenesis (in part by increasing HIF-1α expression), cell invasion and glioblastoma progression in mouse models [27, 34, 35]. Interestingly, in the UCHL1 KDs we found downregulation of additional proangiogenic genes such as *ELK3*, *CXCL12*, *ANGPT1*, *HMOX1*, or *DLK1* and upregulation of the anti-angiogenic gene *THBS1*. UCHL1 has been shown to increase metastasis through deubiquitination and stabilization of HIF-1α, a central protein in tumor angiogenesis [17]. Thus, a positive feedback loop between UCHL1, HIF-1α, and POSTN might be proposed where UCHL1 may promote angiogenesis directly *via* HIF-1α stabilization and indirectly *via* POSTN upregulation.

Additional mechanisms mediating self-renewal and invasion in our model system such as activation of Akt and Erk1/2 pathways [30, 36], or stabilization of the oxidase NOX4 and of membrane receptors like the cell adhesion protein NCAM among other yet to uncover targets [37, 38] should not be ruled out. For instance aberrant expression of the brain aquaporin 1 (AQ1) and 4 (AQ4), which regulate water flow in and out of the cells, have been found in glioblastoma sphere cultures and their role in this tumor has just started to be elucidated [39, 40]. Of note, *AQ4* was the second most downregulated gene in the UCHL1 KDs (~37-fold reduction compared to controls), thus consistent with a possible function of UCHL1 in glioma CSCs. Nevertheless, UCHL1 is highly expressed in the brain and retina, and inactivation of this protein is associated with neurodegenerative diseases such as Alzheimer and Parkinson’s disease [41–44]. Thus, the UCHL1-KD associated transcriptome may be able to uncover cancer associated genes, but also additional genes not involved in cancer *per s*e but rather be considered within the context of neuroprotective functions associated with UCHL1. For example, the observed downregulation in *ABCA4*, a gene encoding a member of the of the ABC family of transporters that is expressed in retinal photoreceptors and its defect causes retinal degeneration such as Stargardt’s disease (reviewed in [45]).

Finally, within the list of upregulated genes in the UCHL1 KD we also found several cancer-associated genes such as *SERPINB4*, *FGF21*, *NUPR1*, *SBSN*, *GDF1*, *HMOX1*, or *SOCS2*, which suggested the activation of potential compensatory mechanisms in these KDs cells. Future work will evaluate whether these genes exert a biological effect in our model system. Nevertheless, the precise mechanisms driving UCHL1 de-regulation in glioma cells and whether the UCHL1 aberrant expression can influence the progression from low to high-grade glioma remains unknown.

In summary, our data indicated that UCHL1 might be an important factor in pediatric high-grade glioma progression, by sustaining the cancer stem cell population. The potential role of UCHL1 in modulating pathways involved in the niche of glioma CSCs such us angiogenesis, extracellular matrix-receptor interaction, or receptor endocytosis and intracellular trafficking, may point to promising targets for treatment in pediatric high-grade gliomas by either targeting directly UCHL1 or its associated pathways.

## Conclusion

Our *in vitro* functional studies and gene expression data demonstrated that UCHL1 regulates glioblastoma CSCs populations and suggested wnt as a potential pathway mediating UCHL1 activity. Angiogenesis, extracellular matrix-receptor interaction, and endocytosis were also identified as processes associated to the UCHL1-KD transcriptome. Given the relevance of the cancer microenvironment in sustaining the CSC niche, future work will aim to explore UCHL1 as a potential mediator of the interactions between glioma cancer cells and the tissue microenvironment and to explore UCHL1 as a prospective therapeutic target for high-grade pediatric glioma.

## Methods and materials

### Cell lines

SJ-GBM2 was obtained from the Children's Oncology Group (COG) Cell Culture and Xenograft Repository. SF188 was kindly provided by Dr. Daphne Haas-Kogan (University of California at San Francisco, USA) and Res186 by Dr. Michael Bobola (University of Washington, Seattle, WA, USA). SJ-GBM2 cells were maintained in Iscove’s Modified Dulbecco’s Medium with 20% Fetal Bovine Serum (FBS; Atlanta^®^ Biologicals, Inc., Lawrenceville, GA), 4mM L-Glutamine, and 1X ITS (5 μg/mL insulin, 5 μg/mL transferrin, 5 ng/mL selenous acid) and 1% penicillin/streptomycin/amphotericin B (Life Technologies, Carlsbad, CA). SF188 and Res186 cells were maintained in DMEM/F12 Ham’s medium (Mediatech, Inc., Manassas, VA, USA) supplemented with 10% FBS and 1% antibiotics. All cells were grown at 37°C in a humidified 5% CO_2_ incubator. Cells were regularly checked for mycoplasma contamination using MycoAlert^™^ Mycoplasma Detection Kit (Lonza, Anaheim, CA). All the experiments shown here were performed with cells free of mycoplasma contamination.

### UCHL1 knockdown generation

Stable UCHL1 knockdowns (KDs) were obtained through a lentiviral transduction system (Addgene) using puromycin (2 μg/mL) as marker for selection. Viral packaging and titration were performed as per Addgene’s standard protocols. Two different shRNA constructs targeting different sequences within the UCHL1 coding region were used (KD1; TRCN0000011079, NM_004181.3-400s1c1, and KD2; TRCN0000007276, NM_004181.3-236s1c1). UCHL1 KD efficiency was confirmed at the mRNA and protein levels (see RT-PCR and western blot sections below for details) and compared to control cells infected with a shTurboGFP vector.

### Western blotting

For western blot analysis, protein was isolated and then quantified using the Bradford assay. Briefly, cells were disrupted in CelLytic (Sigma-Aldrich, St. Louis, MO) buffer. Twenty μg of protein were solubilized in NuPAGE^®^ Sample Reducing Agent and NuPAGE^®^ LDS Sample Buffer (Life Technologies, Carlsbad, CA). Protein extracts were run in precast NuPAGE^®^ 4–12% Bis-Tris gels (Life Technologies) and transferred to nitrocellulose membrane (Life Technologies) using the semi-dry iBlot system (Life Technologies). Membranes were stained with Ponceau Red (Sigma-Aldrich) to confirm protein transfer, washed with 0.1% Tween 20/1x TBS and then were blocked in 0.1% Tween 20/1x TBS with 5% w/v nonfat dry milk overnight at 4°C. After three washes with 0.1% Tween 20/1x TBS blots were incubated overnight at 4°C in 0.1% Tween 20/1x TBS containing 5% w/v nonfat dry milk and antibodies of interest; polyclonal anti-UCHL1 (AB_2210628; 1:5,000 dilution factor; Cell Signaling, Boston, MA), and polyclonal anti-β-Actin (AB_956497; loading control; 1:12,000 dilution factor, Abcam, Inc.) following manufacturers recommendations. After washing with 0.1% Tween 20/1x TBS, membranes were incubated with goat anti-rabbit IgG HRP (AB_10679812; 1:15,000 dilution factor; Abcam, Inc.) conjugated to secondary antibody for one hour at room temperature. Blots were developed using SuperSignal^®^ West Pico Chemiluminescent Substrate detection kit (Thermo Fisher Scientific Pierce Protein Biology Products, Rockford, IL). Band density was measured using a G-BOX CHEMI imaging system (Syngen, Sacramento, CA). Three independent experiments were performed, and one-way ANOVA was used to determine statistical significance using *p* = 0.05 as cutoff value for statistical significance.

### Cell doubling time determination

To determine if UCHL1 was involved in the proliferation of glioma cells we measured the population doubling times (PDT) in SF188 and SJ-GBM2 control and UCHL1 knockdown cells. Briefly, cells were seeded on a 6-well plate (20,000 cells/well) and the number of viable cells counted 2, 4, and 6 days post-plating using trypan-blue exclusion assay on trypsinized cells. The PDT for each cell line was then determined using the formula *PDT = (t-t*_*0*_*)/(log(n) -log(n*_*0*_*))/log(2)*; where *(t-t*_*0*_*)* indicates the time in culture; *n* indicates the number of cells at a given time; and *n*_*0*_ the number of cells plated. Two independent experiments were performed in duplicates.

### Cell cycle analyses

SF188 and SJ-GMB2 control and UCHL1 KDs, cells were synchronized in G0/G1 by serum removal (24-hour treatment) in order to study the kinetics of cell cycle progression. Synchronized cells were then incubated over-night in the presence of complete medium followed by ethanol fixation and propidium iodine staining using standard protocols. Flow cytometry data was obtained using a FACS Canto II, (BD Biosystems, San Jose, CA) and analyzed using MoFlo software. At least three independent experiments with duplicates were performed.

### Clonogenicity assays

Anchorage-independent growth was assessed in vitro using CytoSelect^™^ 96-Well Cell Transformation Assay, Soft Agar Colony Formation (Cell Biolabs, Inc., San Diego, CA) as per manufacturer’s guidelines. One of the main advantages of this method is that colony formation can be quantified with fluorescence thus giving more accurate results than manual colony count. The high sensitivity of this method enables reduced incubation times (at least a 2-fold change) compared to conventional manual methods. Briefly, the wells of the microplate were coated with a 1.2% agar solution. Once this agar layer solidifies, cell suspensions prepared in 0.6% agar-containing medium were added to the microplate wells. After 10 days of incubation, cells were lysed and their DNA (indicator of cell proliferation) stained with CyQuant GR Dye. Anchorage-independent growth was then determined in control and UCHL1 KD cells with a fluorescent plate reader (GloMax II, Promega Madison, WI) using a 485/520 nm filter set.

### Cell invasion assay

The invasive potential of SJ-GBM2 and SF188 control and UCHL1 KD cells was tested *in vitro* using CytoSelect 24-Well Cell Invasion Assay (Cell Biolabs, Inc., San Diego, CA) as per manufacturer’s guidelines. Briefly, cells were seeded in invasion medium, 10% FBS (Atlanta Biologicals, Lawrenceville, GA) and DMEM (Life Technologies, Carlsbad, CA) at 5 x 10^4^ cells/chamber in a 24-well plate. Cells were incubated for 48 hours at 37°C in 5% CO_2_. Non-invading cells were removed with sterile cotton swabs. The cells that were able to migrate through the membrane were then fixed using ethanol, stained with hematoxylin (Sigma-Aldrich St. Louis, MO), and counted under 40x magnification. At least eight fields were counted per well from two independent experiments.

### *In vitro* surrogate assay for CSCs

We used a well-defined protocol [21], to generate spheroid cultures from glioblastoma cell lines in serum-free Neurobasal medium (Invitrogen, Carlsbad, CA) and in the presence basic fibroblastic (bFGF) and epidermal (EGF) growth factors. To evaluate self-renewal, individual 1^st^ generation spheres were harvested, mechanically dispersed into single-cell suspensions and 8 x 10^5^ of these cells plated onto 100 mm diameter ultra-low attachment plates (Corning^®^, Manassas, VA) containing 10 mL of serum-free Neurobasal medium (Neurobasal^®^-A Medium +B27 and N-2 supplements, 2mM L-glutamine, 10 ng/mL bFGF and EGF, and 0.005% BSA; Invitrogen, Carlsbad, CA) to generate second and third generation of spheres. Fresh growth factors were added every three days to the spheroid cultures. All the experiments described here were performed using third generation of spheres. For the neurosphere limiting dilution assay, spheres from the second generation of control and UCHL1 knockdowns were dispersed and re-plated in ultra-low attachment 96-well plates using serial limiting dilution (1 to 200 cells/well range). Third generation sphere formation was scored in each well after 10 days of incubation to provide the optimal cell densities. All subsequent experiments were initiated by plating 2000 cells/mL from dispersed second generation spheres in ultra-low attachment 96-well plates. The number of third generation spheres with a diameter of at least 50 μm was scored after seven days in culture. Three experiments with triplicates were performed.

### TOPflash reporter activity assay

We used a TOPflash luciferase reporter assay to measure the effect of UCHL1 on the Wnt/Beta-catenin pathway activity. Briefly, SJ-GBM2 cells infected with either the UCHL1 shRNA or control plasmids were plated on a 96-well plate (5,000 cells/well). Cells were allowed to recover from the trypsin treatment for 12–18 hours and then transfected with the Renilla luciferase plasmid pRL-TK (Addgene, Cambridge, MA) and either the TOPflash or the FOPflash (mutant TCF/LEF binding sites; control) plasmid using Lipofectamine2000 transfection reagent (Invitrogen, Carlsbad, CA) as per manufacturer’s guidelines. Mock transfections were included as baseline during luminescence analyses. Forty-eight hours post-transfection the Wnt ligand Wnt3a (beta-catenin activator; R&D Systems, Minneapolis, MN) was added to all wells and, 12–18 hours later, the activity of the luciferase reporter activity measured using the Dual-Glo Luciferase Assay System and GloMax-Multi 96-well Microplate reader (Promega, Madison, WI) using Renilla luminescence as control and as per manufacturer’s instructions. Three independent experiments were performed in duplicates.

### Quantitative RT-PCR (qRT-PCR)

Total RNA was extracted from the cells using a phenol-free total RNA extraction kit (Norgen Biotek, ON, Canada). Quality and concentration of the extracted RNA was determined by spectrophotometric methods (NanoDrop 8000 UV-Vis Spectrophotometer, Thermo Scientific, Waltham, MA). The levels of expression of the *UCHL1* and housekeeping gene (*HPRT1*) were measured in the glioma cell lines and in the UCHL1 KDs using droplet digital PCR (QX100 ddPCR System, Bio-Rad, Hercules, CA) and Taqman Gene Expression primer/probes sets (Human *HPRT1* (FAM-labeled) and *UCHL1* (VIC-labeled); Applied Biosystem, Foster City, CA). RT reactions were conducted on a Veriti PCR System (Applied Biosystems) using 100 ng of total RNA, and iScript Reverse Transcription Supermix for RT-qPCR (Bio-Rad) as per manufacturer’s guidelines (i.e. 5 min at 25°C, 30 min at 42°C, 5 min at 85°C followed by an end cycle at 4°C). Quantitative PCR reactions were prepared using 2 μL of the RT reactions (diluted 1/10), Taqman Gene Expression Assays, and ddPCR Supermix for probes (Bio-Rad) as per manufacturer’s guidelines. Differences in *UCHL1* mRNA expression across samples were determined using QX100 Droplet Reader and QuantaSoft TM Software (Bio-Rad).

### Next generation sequencing and ingenuity pathway analyses

Total RNA was extracted using the RNeasy Mini kit (Qiagen, Valencia, CA). Briefly, cells were lysed with Buffer RLT and homogenized using a syringe. RNA was isolated in a spin column and treated with DNaseI. RNA was eluted with DNase and RNase free water. Integrity checks (measured as RNA Integrity Number; RIN) and sample quantitation was performed using the Agilent 2100 Bioanalyzer (Agilent Technologies, Santa Clara, CA).

RNAseq was performed at GCCRI Genome Sequencing Facility. In short, mRNA was isolated by oligo-dT purification and fragmented using divalent cations under elevated temperature. cDNA fragment libraries were synthesized following the TruSeq mRNA-seq Library Preparation protocol (Illumina, San Diego, CA), and sequenced with an Illumina HiSeq 2000 system using a 100 bp paired-end sequencing protocol. On average, we obtained ~35 million paired reads per sample. After sequencing, sequence reads were aligned to human genome (UCSC hg19) using TopHat aligner [46] then reads aligned to known transcripts were counted using HTSeq [47]. Differential gene expression analysis was performed using DESeq [48] to obtain fold-change, *p-*value, and adjusted *p*-value by Benjamini-Hochberg correction for multiple tests. We selected differentially expressed genes based on the following criteria: 1) fold-change > 2, 2) adjusted *p*-value < 0.05, and 3) expression level RPKM (Read Per Kilobase of transcript per Million reads mapped) > 1. A total of 306 genes were selected based on these criteria. Functional assessment of differentially expressed genes was performed by using Database for Annotation, Visualization and Integrated Discovery (DAVID, http://david.abcc.ncifcrf.gov/) and Ingenuity pathway analysis (IPA, Qiagen, http://www.ingenuity.com) were conducted to identify UCHL1-associated gene networks.

### Statistical analysis

Statistical analysis was performed using GraphPad Prism (GraphPad Software version 5.0c). The data were displayed as means ± standard error of the mean (SEM). One-way ANOVA was used to analyze all the data from the in *vitro* assays. Results were considered significant when the *p*-value was equal to or below 0.05.

## Supporting information

S1 File UCHL1 knockdown-associated transcriptome. Three hundred and six differentially expressed genes (adjusted *p-*value < 0.05) were identified in the two UCHL1 knockdown constructs compared to control SJ-GMB2 cells.
(XLSX)

S2 File DAVID cluster analysis UCHL1-associated transcriptome. Eleven annotation clusters with enrichment scores above 2.5 were identified by gene ontology (GO) analysis.
(XLSX)

S3 File IPA analysis summary. Inflammation, endocytosis, metastasis related functions and “High Mobility Group Box-1 (HMGB1) Signaling” were identified within the top canonical pathways associated to the UCHL1 knockdowns.
(PDF)
