# Supplementary material for: Ubiquitin carboxyl-terminal esterase L1 (UCHL1) is associated with stem-like cancer cell functions in pediatric high-grade glioma
Source: PLoS One. 2017 May 4;12(5):e0176879. doi: 10.1371/journal.pone.0176879 (PMC5417601; doi:10.1371/journal.pone.0176879)
Supplement: S3 File — Inflammation, endocytosis, metastasis related functions and “High Mobility Group Box-1 (HMGB1) Signaling” were identified within the top canonical pathways associated to the UCHL1 knockdowns. (PDF) [file pone.0176879.s003.pdf]

INGENUITY®
PATHWAY ANALYSIS

Analysis Name: SJGBM2. Ctrl_vs_UCHL1_KD_result_DEseq_filtered_by pvalue - 2016-07-21 04:32 PM
Analysis Creation Date: 2016-07-21
Build version: 389077M
Content version: 27821452 (Release Date: 2016-06-14)

Analysis Settings

Reference set: Ingenuity Knowledge Base (Genes Only)

Relationship to include: Direct and Indirect

Does not Include Endogenous Chemicals

Optional Analyses: My Pathways My List

Filter Summary:

Consider only relationships where

confidence = Experimentally Observed OR High (predicted)

(c) 2000-2016 QIAGEN. All rights reserved.

Top Canonical Pathways

Name	p-value	Overlap
Role of Macrophages, Fibroblasts and Endothelial Cells in Rheumatoid Arthritis	3.68E-05	4.9 % 15/309
Clathrin-mediated Endocytosis Signaling	5.14E-04	5.1 % 10/197
Virus Entry via Endocytic Pathways	6.15E-04	6.9 % 7/102
HMGB1 Signaling	6.15E-04	6.0 % 8/133
Colorectal Cancer Metastasis Signaling	8.18E-04	4.5 % 11/247

Top Upstream Regulators

Upstream Regulator	p-value of overlap	Predicted Activation
FGF2	3.57E-18	
IL1B	7.67E-18	Activated
TNF	1.04E-16	Activated
PDGF BB	1.95E-16	
dexamethasone	3.82E-16	

Top Diseases and Bio Functions

Diseases and Disorders

Name	p-value	#Molecules
Cancer	1.29E-04 - 5.38E-14	285
Organismal Injury and Abnormalities	1.29E-04 - 5.38E-14	287
Cardiovascular Disease	1.08E-04 - 3.30E-12	73
Neurological Disease	1.22E-04 - 3.41E-12	126
Gastrointestinal Disease	1.29E-04 - 1.11E-11	256

Molecular and Cellular Functions

(c) 2000-2016 QIAGEN. All rights reserved.

INGENUITY PATHWAY ANALYSIS

Name	p-value	#Molecules
Cellular Growth and Proliferation	1.17E-04 - 5.84E-22	175
Cellular Development	1.43E-04 - 5.63E-19	154
Cellular Movement	1.24E-04 - 7.92E-16	108
Cell Death and Survival	1.31E-04 - 6.47E-15	128
Cell Morphology	1.22E-04 - 2.07E-14	118

Physiological System Development and Function

Name	p-value	#Molecules
Organismal Survival	1.76E-06 - 1.38E-15	110
Connective Tissue Development and Function	9.46E-05 - 3.02E-15	93
Tissue Development	1.43E-04 - 3.02E-15	160
Cardiovascular System Development and Function	1.15E-04 - 4.83E-15	87
Organismal Development	1.41E-04 - 2.90E-14	153

Top Tox Functions

Assays: Clinical Chemistry and Hematology

Name	p-value	#Molecules
Increased Levels of Albumin	1.08E-01 - 1.41E-02	3
Increased Levels of Alkaline Phosphatase	1.90E-02 - 1.41E-02	5
Increased Levels of Red Blood Cells	5.50E-02 - 5.50E-02	4
Decreased Levels of Albumin	1.69E-01 - 6.87E-02	2
Decreased Levels of Hematocrit	1.20E-01 - 1.20E-01	1

Cardiotoxicity

(c) 2000-2016 QIAGEN. All rights reserved.

INGENUITY PATHWAY ANALYSIS

Name	p-value	#Molecules
Cardiac Infarction	1.57E-01 - 6.60E-06	16
Cardiac Hypertrophy	3.49E-01 - 3.18E-05	18
Cardiac Arteriopathy	1.08E-01 - 1.08E-04	15
Cardiac Necrosis/Cell Death	3.75E-01 - 2.26E-04	12
Cardiac Damage	1.14E-01 - 3.11E-04	6

Hepatotoxicity

Name	p-value	#Molecules
Liver Proliferation	5.09E-01 - 5.64E-05	12
Liver Hyperplasia/Hyperproliferation	5.09E-01 - 3.68E-04	119
Liver Damage	6.04E-01 - 7.10E-04	14
Liver Necrosis/Cell Death	2.58E-01 - 8.10E-04	12
Liver Fibrosis	5.09E-01 - 9.45E-04	10

Nephrotoxicity

Name	p-value	#Molecules
Kidney Failure	2.58E-01 - 1.09E-04	13
Renal Inflammation	1.99E-01 - 5.91E-04	8
Renal Nephritis	1.99E-01 - 5.91E-04	8
Renal Damage	1.92E-01 - 8.00E-04	11
Glomerular Injury	1.92E-01 - 3.52E-03	9

Top Regulator Effect Networks

ID	Regulators	Diseases & Functions	Consistency Score
1	DICER1,EBF1,ERBB4,MAP3K8,NFATC2	development of lymphocytes (+2 more)	7.75
2	F2,IL10RA,ITGB1,NEDD9,NEUROG1,SP1	microtubule dynamics	5.578

(c) 2000-2016 QIAGEN. All rights reserved.

INGENUITY PATHWAY ANALYSIS

3	ERBB4,IL3,TP53	fibrosis of liver	3.402
4	CNTF,MAP3K8,MAPK9,NFATC2	differentiation of lymphocytes	3.333
5	INHA,SP3	mitogenesis	2.236

Top Networks

ID	Associated Network Functions	Score
1	Endocrine System Disorders, Immunological Disease, Inflammatory Disease	42
2	Cell Death and Survival, Lipid Metabolism, Molecular Transport	35
3	Cellular Growth and Proliferation, Immunological Disease, Inflammatory Disease	33
4	Digestive System Development and Function, Embryonic Development, Organ Development	33
5	Digestive System Development and Function, Connective Tissue Disorders, Developmental Disorder	28

Top Tox Lists

Name	p-value	Overlap	
Acute Renal Failure Panel (Rat)	2.52E-06	12.9 %	8/62
Cardiac Hypertrophy	5.16E-05	4.1 %	18/435
Liver Proliferation	1.03E-04	5.3 %	12/228
Cardiac Necrosis/Cell Death	5.38E-04	4.4 %	12/273
Liver Necrosis/Cell Death	8.86E-04	4.2 %	12/289

Top My Lists

Name	p-value	Overlap	
CFC_MMS_YO_Cancer	2.66E-09	12.5 %	13/104
Inflammation	5.52E-09	3.3 %	53/1608
MRU_MMS_YO_cancer	1.90E-05	11.9 %	7/59
Oxidative Metabolism	1.73E-02	3.5 %	8/231
Mitochondria	1.84E-02	2.7 %	13/476

(c) 2000-2016 QIAGEN. All rights reserved.

Top My Pathways

Name	p-value	Overlap
let-7	1.23E-02	3.7 % 8/217
mTOR Signaling	2.33E-02	3.5 % 7/199
TGF- Signaling	1.25E-01	3.4 % 3/87
ERK/MAPK Signaling	1.52E-01	2.5 % 5/199
p38 MAPK Signaling	2.29E-01	2.6 % 3/117

Top Analysis-Ready Molecules

Exp Log Ratio up-regulated

Molecules	Exp. Value	Exp. Chart
**SERPINB4**	↑ 5.957	
**TAC1**	↑ 5.432	
**FGF21**	↑ 4.622	
**ANKRD1**	↑ 4.197	
**ENPP2**	↑ 4.137	
**SBSN**	↑ 4.054	
**GDF15**	↑ 3.737	
**MEIOB**	↑ 3.703	
**NGFR**	↑ 3.643	
**MT1G**	↑ 3.566	

Exp Log Ratio down-regulated

Molecules	Exp. Value	Exp. Chart
**IGLL1/IGLL5**	↓ -5.329	
**AQP4**	↓ -5.213	
**ABCA4**	↓ -4.766	

(c) 2000-2016 QIAGEN. All rights reserved.

**GRAP2**	↓ -4.748
**FAM83F**	↓ -4.457
**SP7**	↓ -4.239
**CALB1**	↓ -4.188
**PTPN22**	↓ -3.940
**TAC3**	↓ -3.854
**PDE7B**	↓ -3.764

(c) 2000-2016 QIAGEN. All rights reserved.

INGENUITY PATHWAY ANALYSIS
